# Consumer behavior and its role in *E. coli* outbreaks: the impact of fast-food preparation practices and hygiene awareness

**DOI:** 10.1186/s41182-025-00710-y

**Published:** 2025-02-24

**Authors:** Victor Oluwatomiwa Ajekiigbe, Ikponmwosa Jude Ogieuhi, Chidera Stanley Anthony, Ifeoluwa Sandra Bakare, Sopuruchukwu Anyacho, Praise Oluwatobi Ogunleke, Damilola Ifeoluwa Fatokun, Olufemi Akinmeji, Osineye Tolulope Ruth, Akintomiwa Kolawole Olaore, Oluwafemi Amusa, Chinonyelum Emmanuel Agbo

**Affiliations:** 1 Ladoke Akintola University of Technology, Ogbomoho, Nigeria; 2 Siberian State Medical University, Tomsk, Russia; 3 University of Calabar, Calabar, Nigeria; 4 Ternopil State Medical University, Ternopil, Ukraine; 5 University of Ilorin, Ilorin, Kwara State Nigeria; 6 Olabisi Onabanjo University, Sagamu, Nigeria; 7 University of Nigeria, Nsukka, Nigeria

**Keywords:** *E. coli*, Fast food, Consumer behavior, Diarrhea, Foodborne diseases

## Abstract

**Background:**

The fast-food industry, a rapidly expanding business due to the influence of urbanization and busy lifestyles, has significantly shaped consumer food habits and quality food-seeking behavior. However, this fast-growing sector is frequently challenged by bacteria of clinical, microbiological, and economic importance, including *Escherichia coli (E. coli)*. While many strains of *E. coli* are harmless and support digestion, pathogenic variants such as *E. coli* O157:H7 are responsible for severe foodborne illnesses, public health crises, and economic losses.

**Main body:**

Our study explores consumer behavior within the fast-food industry, highlighting its role in shaping responses to *E. coli* outbreaks. Also, it examines how increased awareness of food safety risks has influenced consumer decisions, such as adopting hygienic practices and preferring establishments that prioritize food safety. Furthermore, the study investigates the contribution of poor fast-food preparation practices—such as undercooking and cross-contamination—to the spread of *E. coli* and emphasizes the critical need for improved hygiene awareness among fast-food workers. We analysed notable case studies involving *E. coli* outbreaks linked to fast-food chains, and subsequently identified gaps in industry practices and consumer behavior that exacerbate the risk of foodborne illnesses. This emphasizes the importance of preventive measures, including industry-driven reforms such as enhanced food handling protocols and consumer education programs, to mitigate future outbreaks.

**Conclusions:**

This study aims to provide evidence-based insights into the shared responsibility of fast-food establishments and consumers in reducing the prevalence of *E. coli* infections. By addressing gaps in hygiene awareness and preparation practices, the findings emphasize the potential for collaborative efforts to strengthen public health outcomes and prevent further outbreaks.

## Background

### Overview of *E. coli* as a foodborne pathogen

*Escherichia coli* (*E. coli*) is a Gram-negative, facultative, anaerobic, non-spore-forming, rod-shaped bacterium belonging to the Enterobacteriaceae family and the enteric genus Escherichia and its close phylogenetic relative, Shigella [1]. Although the genus Escherichia includes six species, *E. coli* is the commonest of the bacterial species most frequently isolated in the clinical microbiology laboratory. It is a commensal organism that forms part of the normal flora of the intestinal tracts of humans and animals [2, 3].

*E. coli* strains are classified according to the ‘O-antigen’ found in the lipopolysaccharide of the outer cell envelope, as well as the flagellar ‘H-antigen’ and the capsular ‘K-antigen’ [4], with the combination of O- and H-antigen defining the *E. coli* serotype. Most of the serotypes of *E. coli* are harmless and play a crucial role in food digestion [5]. However, some variants have acquired different virulence genes, enabling them to cause various diseases, commonly foodborne illnesses [6, 7]. Some pathogenic *E. coli* causes diarrhoea and other intestinal diseases, while others cause disease at non-intestinal sites, including pneumonia, bacteremia, and spontaneous bacterial peritonitis [8, 9].

Six major foodborne diarrheagenic *E. coli* (DEC) pathotypes are classified based on their virulence factors, attachment patterns to host cells, effects of this attachment, toxin production, and invasiveness. These pathotypes include Enteropathogenic *E. coli* (EPEC); Shiga toxin-producing *E. coli* (STEC), which includes the subgroup Enterohemorrhagic *E. coli* (EHEC); Shigella/Enteroinvasive *E. coli* (EIEC); Enteroaggregative *E. coli* (EAEC); Enterotoxigenic *E. coli* (ETEC); and Diffusely Adherent *E. coli* (DAEC) [2].

Food contamination by pathogenic microorganisms has been a significant public health concern and has caused global economic losses. The World Health Organization (WHO) reported that the *E. coli* O104:H4 outbreak in 2011 led to a $1.3 billion loss for farmers and industries in Germany and necessitated $236 million in emergency aid to 22 member states of the European Union [10, 11]. Notably, the O104:H4 serotype responsible for this outbreak was an unusual and novel pathotype. It exhibited features typical of EAEC while also carrying Shiga toxin-producing genes, making it a highly virulent strain [10]. Foodborne contamination with pathogenic *E. coli*, including strains like O157 and O104, is quite prevalent, even in developed countries [12]. *E. coli* can be transmitted from one person to another through the fecal–oral route and via contaminated food, unchlorinated water, raw milk, and improperly cooked beef [13].

On September 27, 2024, a major *E. coli* outbreak associated with McDonald's was reported [14]. By December 3, the Centers for Disease Control and Prevention (CDC) confirmed that at least 104 people in 14 states across the United States became ill after eating Quarter Pounders from McDonald's [14]. Among those affected, 34 required hospitalizations, with 4 experiencing severe kidney complications that could result in permanent damage or death. Additionally, one fatality was reported from an older adult in Colorado. The CDC identified slivered onions used in the Quarter Pounders as the likely source of contamination [14].

There have been several significant outbreaks in the past, especially those linked to STEC O157:H7, which have a very low infectious dose (10–100 cells), significantly increasing the risk of outbreaks, which have been associated with various food sources, including undercooked ground beef, raw milk, salads, raw leeks, lettuce, spinach, potatoes, and other vegetables [14, 15]. In 1982, two outbreaks of severe bloody diarrhoea syndrome occurred in Oregon and Michigan, linked to the consumption of fast-food hamburgers [15]. A severe outbreak happened in 1993 when *E. coli* O157:H7 contaminated undercooked ground beef sold at Jack in the Box restaurants in the Pacific Northwest [16]. Notable reforms to food safety regulations and practices were made after children were affected by the outbreak, particularly ground beef processing. A major outbreak was encountered by Germany in 2011, which was associated with contaminated sprouted seeds, specifically fenugreek roots. This culminated in 4,075 reported cases and 50 deaths in Europe transversely. This led to the identification of the *E. coli* strain O104:H4, which exhibited characteristics of both *E. coli* O157 and enteroaggregative *E. coli* (EAEC) [11, 17].

Another notable outbreak occurred in 2018, connected to romaine lettuce from the Yuma, Arizona area [18]. This incident traced the *E. coli* O157:H7 strain to canal water contaminated by runoff from cattle feedlots upstream, underscoring the complex routes through which contamination can affect fresh produce. In 2020, an outbreak in the US linked to fresh leafy greens was reported, resulting in 40 confirmed cases, 20 major hospitalizations and 4 Hemolytic Uremic Syndrome (HUS) [18–20]. The investigation showed that multiple leafy greens were involved, illustrating how various products can be implicated in *E. coli* outbreaks [19].

### The link between *E. coli* outbreaks and consumer behavior

The *E. coli* outbreak has been a major eye-opener regarding how health catastrophes can impact the decision-making process in food procurement, safety, and trust in food brands [21]. Consumers regularly reform their shopping habits following such episodes. For instance, after the popular *E. coli* outbreak associated with spinach, the level of distrust in consumers of freshly produced items soared, thereby making consumers opt for pre-packaged or processed foods that appear safer. They exemplify how the fear of contamination might make consumers more careful in their choices on a larger scale [22].

Furthermore, outbreaks of *E. coli* often make consumers more vigilant about food safety. Consumers tend to be more mindful of dangers embedded with certain foods, yielding more attention to food labels and procurement etiquette. The drift in information-seeking patterns by consumers about the origins and handling of their foods was triggered by the 2018 outbreak related to romaine lettuce [23].

The roles played by food brands and retailers amid an outbreak speak volumes and are pivotal in influencing consumer behaviors. Proper management of outbreaks by brands—through issuing quick product recalls and keeping open and lucid communication—has a greater tendency to retain consumer loyalty. Conversely, poor responses by the brands can disdain their reputation to loyal and potential consumers of their products. More recently, the *E. coli* outbreak associated with McDonald’s illustrates how disastrous it could be if consumer trust is lost, leading to low sales returns and a remarkable fall in their stock value.

Beyond immediate reactions, *E. coli* outbreaks can result in enduring changes in consumer behavior. As consumers become more informed, they are more inclined to adopt safe practices, such as washing their hands before meals, choosing establishments emphasizing food safety, and being cautious about cross-contamination. Studies show that many individuals may avoid certain foods or brands even after the immediate danger has passed [24, 25]. This trend was clear following the 2018 romaine lettuce and 2006 spinach outbreak, during which consumers were reluctant to buy these produce for a prolonged time [23, 26]. Another notable event was the 2015 Chipotle incident, which led to a decline in customer visits and 74% of surveyed consumers expressing a demand for improved food safety monitoring in restaurants [27].

Consumer actions can influence food safety regulations in the long run, and heightened public awareness of foodborne illnesses can spur compelling advocacy for measures in food safety, stimulating regulatory agencies to institute stringent guidelines. This became apparent after several phenomenal outbreaks, where the consumers' urge for safer food practices has resulted in significant legislative changes [28].

## Main text

### Consumer behavior in the context of fast foods

#### Demand for convenience

Today, the demand for convenience has emerged as a key aspect of consumer behavior, especially within the fast-food sector. As people lead increasingly busy lives and time becomes more valuable, many look for quick and accessible meal options. In recent decades, this has increased the popularity of out-of-home food, such as takeaway, take-out, and fast food [29]. Fast food refers to readily prepared meals designed for quick service and consumption, often featuring processed or pre-cooked ingredients [30]. Examples include burgers, fried chicken, pizza, hot dogs, hamburgers, sandwiches, fries, and sugary beverages [31].

Findings from the Heckman model indicate that household size, education level, and the distance from the workplace to the restaurant negatively impacted the likelihood of consuming fast food and the amount spent on it [32]. The typical preparation time required for home-cooked meals can be overwhelming for those with busy lifestyles. In contrast, fast food restaurants offer a quick solution to hunger, allowing customers to eat within minutes [33].

The evolution of technology has not only ameliorated convenience but also influenced consumers' actions by increasing the accessibility to fast food. Consumers' engagement with fast food brands has changed due to the advent of mobile apps. Placing an order through an app has averted the arduous stress consumers face in long queues and modified their meals. Moreover, discounts, coupons, and promotions on these apps engage consumers and increase the need for more convenient food options. The use of delivery services has been on the rise and has also impacted on how consumers patronize and enjoy fast foods from their spaces, whether at work or home, with the COVID-19 pandemic playing a significant part in consumer’s behavior, evidenced by a study by Columbia Business School which found that the pandemic alone was responsible for 70% of the industry's growth from 2019 to 2020. This makes the process of food ordering swift by providing orders just at their fingertips [34, 35].

Consumer habits regarding fast food consumption vary significantly across different regions and age groups. For instance, adolescents and young adults are more inclined towards fast food consumption compared to other age groups [36]. Also, rural consumers often prioritize factors like affordability, value for money, convenience, and accessibility when choosing fast food outlets, which may differ from urban consumer preferences [37].

Numerous fast-food chains have introduced healthier options for consumers interested in nutritious choices by adding healthier menu items and providing clear nutritional information. As consumers increasingly look for quick meals that fit their dietary preferences, fast food chains offering healthy and convenient options tend to draw a broader customer base.

#### Perceptions of fast-food safety

In recent years, the perception of food safety has emerged as a critical determinant of consumer choices in the food market [38]. As people become more health-conscious, their food choices are influenced by their perceptions of the safety of those products. Research indicated that many fast-food consumers are worried about food hazards, including pesticide residues on vegetables, the overuse of artificial flavour enhancers and colourants, bacterial contamination, harmful substances leaching from plastic packaging, and the overall unhygienic conditions in which food is prepared and sold [39]. Consumers also raised concerns about foodborne diseases such as cholera, typhoid, diarrhoea, bird flu and swine flu [40].

Various factors, such as individual preferences, social influences, cultural backgrounds, and psychological aspects, affect these behaviours. Personal experiences also significantly shape perceptions; for example, research indicates that consumers often avoid purchasing foods they perceive as unsafe. Even after a safety problem with a particular food has been resolved, consumers’ negative perceptions about the implicated food product and about the ability of the supplier or exporting country to produce safe food may linger [41]. Additionally, knowledge and awareness of food safety practices are crucial; consumers informed about safe food handling and the associated risks are more likely to prioritize safety in their choices [42].

Suggestions from family and friends and information shared on social media greatly influence perceptions of food safety. For example, a peer's warning about a specific brand or product can result in heightened scrutiny and caution. The media also plays a significant role in shaping these perceptions. News coverage of food recalls, illness outbreaks, and studies exposing contamination risks adds to public anxiety. Social media platforms are also breeding grounds for misinformation and pseudoscience, including myths and misconceptions about food safety. Regular exposure to negative information can lead to skepticism about the safety of food systems [43–45]. Furthermore, as consumers become more aware of food safety concerns, their overall health consciousness also rises. This heightened awareness frequently results in a stronger focus on buying organic, local, or minimally processed foods, which are considered safer. However, recent studies indicate that organic produce can be contaminated with pathogens. For instance, a 2023 study found that organic vegetables had a higher contamination risk index (2–4) compared to conventional vegetables (1–2) [46]. Another recent study in a 2024 *E. coli* outbreak in the U.S. was linked to contaminated organic carrots, resulting in 39 illnesses and one death [47].

Consumers are also willing to pay more for products marketed as safe or healthy. This trend highlights the important role that perceived food safety has in influencing preferences and purchasing behavior. In a market filled with options, the guarantee of safety can be a critical factor in decision-making.

### Fast food preparation practices and *E. coli* outbreaks

#### Common fast-food preparation issues

Foodborne diseases are caused by contamination and can occur at any stage of the food production, delivery, and consumption chain. They can result from several forms of environmental contamination, including water, soil, or air pollution and unsafe food storage and processing [48].

WHO estimates that 33 million healthy life years are lost annually due to unsafe food consumption worldwide [49]. Notably, 600 million cases of foodborne diseases and 420,000 deaths occur each year globally [Food safety]. Due to busy lifestyles, modernization, and insufficient time to prepare meals at home, eating out has become common for many people [50, 51]. In Canada, it is estimated that there are about 13,000 foodborne cases of *E. coli* O157 every year, which can sometimes be contracted through leafy greens and ready-to-eat beef from fast food [52]. A more recent study has shown that the situation has changed since then as the government of Canada estimates that there are approximately 11 million cases yearly, a dramatic increase from 2014 [53]

Food safety is crucial in food preparation, including purchasing raw materials, preparation, storage, and distribution. Food deliveries and eating out of homes have become common [40]. In developing countries, inadequate food safety standards contribute to a higher risk of contamination due to limited sanitary infrastructure [54, 55]. A study carried out in Egypt found that raw beef burgers contained *E. coli* O157:H7 in high proportion, which suggests that *E. coli* O157:H7 is likely to contaminate healthy bovine meat during slaughter and processing [55]. However, the safety of these foods and their effect on the health of the consumer could become a concern, if necessary, preparation steps and hygienic practices are not followed during their preparation and distribution [54].

Therefore, it is essential to apply hygienic and sanitary practices at all steps of food production and preparation. Implementing food quality and food safety monitoring programs by food service establishments would dramatically improve food quality and safety [50]. These improvements will be reflected in the degree of the food acceptability served in these restaurants [50]. Global food safety guidelines, such as the WHO and FAO's Codex Alimentarius, provide a framework for national regulatory systems [56]. The primary challenge lies in the lack of enforcement in certain countries. Many developing nations struggle with implementing these standards due to limited resources and infrastructure [56].

### Supply chain vulnerabilities hygiene awareness and its role in preventing *E. coli* spread

The increased fast-food consumption due to urbanization has increased the number of associated foodborne outbreaks. Various stressful conditions along the supply chain, such as temperature fluctuations during storage and transport, even from 4.4 to 10 to 12°C (40 to 50 to 54°F), can stimulate rapid growth of pathogenic and spoilage bacteria such as *E. coli* and *Salmonella* into the food chain, thereby leading to infectious diseases [57, 58]. Bacteria grow most rapidly between 4.4 and 60°C (40 and 140°F), a range often referred to as the "Danger Zone," where they can double in number within a timeframe as short as 20 min [59]. Challenges are faced by fast-food workers, retailers, government agencies, and consumers while ensuring products are safe and will not result in foodborne illness [60].

The major factors responsible for foodborne illnesses are insecure food sources, improper cooking, cross-contamination, food mishandling, poor storage conditions, and poor personal hygiene [54]. Bacteria can form biofilms during food processing, which are bacterial micro-colonies attached to surfaces and composed of various extracellular substances. These biofilms protect bacteria against cleaning agents, allowing them to persist in food processing facilities [57].

### Hygiene practices and awareness in fast food: workers and consumers

Fast food is a popular choice for consumers around the world, but its food safety depends on the hygiene practices of both workers and consumers. Understanding how both groups contribute to food safety is key to reducing the risk of foodborne illness.

### Hygiene awareness among fast food workers

In a study by Sharaf 2020, which was carried out in Jordan, the food handlers showed high knowledge of the sources of food contamination with food pathogens, food with high risk, risk factors for food poisoning, and storage conditions for food [61]. However, they also demonstrated weak knowledge about reheating food previously prepared before eating. *E. coli* strains like O157 can cause severe gastrointestinal abnormalities, such as abdominal cramps and bloody diarrhoea, as well as critical illnesses, such as hemolytic uremic syndrome (HUS) [62].

Most of the fast-food workers in this study by Sharaf 2020 revealed a weak relationship between their knowledge of food hygiene and their practices, which means good knowledge of food safety does not necessarily result in good hygiene practice [61]. Fast food restaurants and cafeterias always assure their customers of freshness, assorted flavours, great and fast delivery service, and discounted prices [63]. However, many fast-food workers do not have sufficient knowledge of food safety and hygiene. Hence, they mostly rely on their wrong experiences and practices, which results in food-borne diseases [55].

However, fast-food workers should have some measures they put in place to ensure the safety of foods and prevent contamination by pathogens, which may include covering food when cooking, cooking and serving food at the right temperature to avoid bacteria contamination and using the correct method for cooking and serving to ensure food safety [63].

Hygienic practices, such as properly washing hands with antiseptic soap under running water and keeping fingernails clean and properly trimmed, should be encouraged to prevent microbial contamination of fast foods [55, 63]. In developed countries, such as the USA, Canada, and Europe, fast-food restaurants typically implement Hazard Analysis Critical Control Point (HACCP) systems and undergo regular sanitary inspections to ensure food safety [64]. In contrast, developing countries often face challenges including inadequate infrastructure for food production, processing, and distribution, as well as limited access to clean water and sanitation facilities [64, 65].

Measures should be implemented to ensure that all fast-food workers have some knowledge of the fast-food business [54]. Food control officers should also ensure that formal food safety education is important to secure safer street fast food [50]. Fast-food workers should be educated on food safety and hygiene to improve the prevention of food poisoning due to microbial contamination. Neatness should also be emphasized, including washing hands properly during food preparation and cleaning kitchen facilities [50].

In terms of the impact of consumer hygiene practices, foodborne illness can be caused by a lack of proper food storage, contamination of cooked and raw foods before eating by contact with pathogen-carrying food or equipment, and bad management of food cooking, which can allow bacteria to survive [63].

Improving food safety programs and strengthening food safety policies, including risk assessment, can enhance food safety, especially in fast-food restaurants [66]. Established programs such as the HACCP offer an organized approach to recognizing and averting potential hazards during food preparation and storage. This program, which was originally developed in the US, and now adopted by many countries, including the European Union and the United Kingdom, emphasizes proactive measures to prevent foodborne diseases, including monitoring critical control points like cooking temperatures and storage conditions, ultimately reducing the burden of food contamination in these countries [67].

Furthermore, United States’ food safety policies, particularly the Food Safety Modernization Act (FSMA), mandate relevant standards to prevent food-related illnesses, including proper staff training, reliable systems to track food sources, and routine inspections of fast-food facilities [68]. Such policy shifts the focus from reactive responses to preventive measures in food safety management. Strengthening the awareness and implementation of these programs and also establishing clear guidelines for hygiene, cleaning protocols, and equipment maintenance, through measures such as Good Manufacturing Practices (GMPs) and Standard Operating Procedures (SOPs), will limit risks of contamination in fast-food production [69, 70].

### Case studies of *E. coli* outbreaks linked to fast-food chains

The fact these aforementioned pathogenic strains of *E. coli* have been implicated in several foodborne outbreaks globally calls for robust and effective food safety measures.

For example, EPEC is frequently linked with outbreaks of diarrheal disease, particularly among children in developing countries, where contamination of water and dairy products is common [71]. Similarly, STEC, including EHEC, has been implicated in a plethora of severe outbreaks worldwide. Notable among them include the European outbreak in 2011, which was traced to fenugreek sprouts, resulting in close to a thousand cases of HUS and more than 50 mortalities in 16 countries [72].

ETEC is often linked to the consumption of contaminated fruits, vegetables, and street vendor-served meals in endemic regions, causing a disease state popularly known as traveler’s diarrhea [73]. While rare EIEC outbreaks affecting all age groups have been traced to contaminated baby spinach, salads and RTE meals [74], EAEC is often responsible for the persistent diarrhea outbreaks among immunocompromised populations, including children, older adults, and HIV-infected individuals [75].

In Washington state, USA, 501 cases of infection with *E. coli* O157:H7 were reported between December 1, 1992 and February 28, 1993. Among these victims, 453 confirmed recently eating hamburgers at a Washington restaurant chain while 48 had secondary infections. This outbreak resulted in 151 hospitalizations due to severe symptoms including bloody diarrhea, 45 cases of HUS, and 3 mortalities. In response to this outbreak, the public health authority withdrew more than 250,000 potentially contaminated hamburgers from the food market, preventing about 800 new cases [76]. In the same year, a major outbreak involving E. coli O157 was reported in multiple cities in the US (Washington, Idaho, California, and Nevada). A total of 603 cases of bloody diarrhoea were confirmed, the majority of which were linked to recent consumption of hamburgers in chain A restaurants. In Washington, 144 of 477 cases were hospitalized for severe illness, 30 were complicated by HUS and 3 died. 34 cases reported in California resulted in 14 major hospitalizations, 7 HUS, and 1 death. This outbreak prompted several measures to strengthen surveillance systems and disease reporting across these cities [77].

Three of the five gastrointestinal sickness cases that the New Brunswick Department of Health reported to the Public Health Agency of Canada on December 31, 2012, were identified as *E. coli* O157 [78]. The Public Health Agency was informed of seven *E. coli* O157 infections in Nova Scotia 2 days later [78]. The patients in Nova Scotia and New Brunswick had closely grouped illness onset dates. In both provinces, all illnesses seemed geographically distributed, community-acquired, and often included fast food chains with 83% of infected individuals reporting recent consumption of sandwiches or burgers garnished with shredded lettuce from chain A or B restaurants [78]. Similar to this outbreak, 29 cases of E. coli O157:H7 infections were reported in 5 provinces of Canada between July 12, 2013 and September 29, 2013. Notably, 26 (90%) of these cases were linked with the consumption of Gouda milk cheese stemming from a dairy facility in British Columbia. This outbreak resulted in 5 serious illnesses and 1 death [79].

On January 4, 2013, public health and food safety partners formed an Outbreak Investigation Coordinating Committee to investigate and manage a nationwide outbreak by the Food-borne Illness Outbreak Response Protocol (FIORP) [80]. Local and provincial public health experts who investigated the outbreak noticed early in the inquiry that a higher percentage of victims indicated exposure to fast food chains in one province, i.e. exposure to “chain A” was recorded often. The fast food and “chain A” targeted questionnaire, centralized follow-up interview, and central exposure data collection and analysis were all employed to support a hypothesis. To get high-quality exposure data, patients were promptly re-interviewed. A thorough history of exposure to fast food restaurants was obtained because of the targeted questionnaires [78].

By using a trace-back analysis of case food histories, The Canadian Food Inspection Agency discovered certain shredded lettuce (iceberg and romaine) that “Importer/Processor X” packaged and delivered to Chains A and B [81]. The Canadian Food Inspection Agency requested, on January 10, 2013, a health risk assessment for the shredded lettuce provided at Chains A and B. Following the evaluation, Health Canada designated a “Health Risk 1” to the identified lettuce, meaning there was a good chance that eating the lettuce might have negative health effects [81]. Even though it was uncertain that any contaminated product would still be accessible due to its short expiration, Importer/Processor X started a precautionary recall for shredded lettuce items supplied to ‘Chain A’ and ‘Chain B’ restaurants that same day. On January 13, 2013, the recall was extended to cover further items made by Importer/Processor X using the same lots of lettuce that were implicated [78].

Another study that investigated the outbreak of STEC in the United Kingdom in August 2020 provided evidence that salad and related items are a significant factor responsible for STEC outbreaks as provided by epidemiological findings which conclusively showed that a particular fast-food product (product X) from restaurant ‘chain A’ was a cause of the infection, i.e. imported cucumbers [82]. In this case, the Food Standards Agency (FSA) coordinated food chain investigations with the outlets identified by the initial epidemiological investigations. The identified fast-food restaurant had previously only provided a restricted menu because of COVID-19 restrictions; however, this associated food product was recently reintroduced into the menu [82].

Because these food products have a limited expiration period, analysing the food items during epidemic findings is sometimes impossible. For this outbreak, (STEC in the United Kingdom in August 2020), the components of the fast-food product X were examined two days before the cucumber batch was distributed, and no STEC infestation was discovered. STEC can be extremely challenging to isolate from associated food products since the infectious dosage is usually lower than that discovered by conventional testing. Hence, such negative microbiological results in associated food products (particularly salad and related components) are not strange [82, 83].

McDonald's has temporarily removed some specific food components, including their Quarter Pounder hamburgers, from many locations due to an *E. coli* outbreak connected to their fast-food restaurant [14]. Multiple federal and state agencies are investigating the outbreak reported on September 27, 2024 [14]. The strain of *E. coli* O157:H7 has infected 104 individuals in 14 states, up from the first reported figure of 49 cases across 10 states [14]. The number of hospitalizations has increased from the 10 first disclosed to 34 [14]. Out of the 81 individuals interviewed thus far, all of them have recalled dining at McDonald's; 80 (99%) of them have reported having a hamburger with beef, 75 have recalled a particular beef hamburger they had there, and 63 (84%) of them have mentioned eating a menu item containing fresh, slivered onions at McDonald's [14].

A few people impacted by this outbreak (*E. coli* outbreak linked to McDonald’s quarter pounders) reported visiting other states before getting sick. During their excursion, not less than three had eaten at McDonald's. McDonald's restaurants have discontinued supplying their current supply of Quarter Pounder beef patties and slivered onions in several states, including Colorado, Kansas, Utah, and Wyoming, as well as portions of Idaho, Iowa, Missouri, Montana, Nebraska, Nevada, New Mexico, and Oklahoma [14].

The United States Department of Agriculture (USDA), Food Safety and Inspection Service, Food and Drug Administration (FDA), and Centers for Disease Control and Prevention (CDC) are working together to identify the source of the contamination through product sampling and distribution data. The impacted McDonald's restaurants receive sliced onions from Taylor Farms, which has voluntarily stopped selling its products [14]. The corporation informed other food chains that may have received potentially contaminated onions [84].

Table 1 summarizes major outbreaks in the past, with emphasis on outbreak date, location, population affected, *E. coli* strain implicated, food type involved, outcomes, interventions and responses.

**Table 1:** Major *E. coli* outbreaks in the past

Outbreak date	Location	Population affected	*E. coli* strain implicated	Food type involved	Outcomes	Interventions and responses
1982 [15]	One in Oregon (February–March) and one in Michigan (May–June)	47 people: 25 from Oregon, 1 from an adjacent county, and 21 from Michigan	*E. coli* O157:H7	Hamburgers from the same fast-food restaurant chain containing three ingredients in common (beef patty, rehydrated onions, and pickles)	Several cases of illness marked by bloody diarrhoea, abdominal cramps, and low-grade fever. Few cases of non-bloody diarrhoea and HUS are recorded	Both victims and neighbourhood controls were interviewed to trace the source of infection Specimens from food samples, blood and stool of victims were investigated in laboratories. Cases were managed with fluid resuscitation and antimicrobials
December 1, 1992–February 28, 1993 [76]	Washington, USA	501 people: 453 ate at a Washington restaurant chain, 48 had secondary infections	*E. coli* O157:H7	Hamburgers	151 (31%) hospitalizations for bloody diarrheal disease, 45 (9%) cases of HUS, and 3 deaths	More than 250,000 potentially contaminated hamburgers were removed from circulation, averting an estimated 800 cases Surveillance system that mandated the reporting of *E. coli* O157:H7 infection was established, prompting outbreak recognition and control. Consumers and food service workers were educated about cooking hamburger meat thoroughly
November 15, 1992–February 28, 1993 (Jack in the Box outbreak) [77]	Multiple states in the US, including Washington, Idaho, California (San Diego), and Nevada (Las Vegas)	603 total reported cases of bloody diarrhea or HUS across many US cities Washington: 477 cases, of which 372 (88%) reported eating at chain A restaurant Idaho: 14 cases, of which 13 (93%) recently ate at a chain A restaurant California: 34 cases, of which 4 were linked to chain A and 4 to chain B restaurants Nevada: 58 cases	*E. coli* O157	In Washington, 312 (92%) ate regular sized hamburger patties	In Washington, 144 were hospitalized for severe illness, 30 developed HUS, and 3 died In Idaho, 4 were hospitalized and 1 developed HUS In California, 14 became hospitalized, 7 were complicated with HUS and 1 died In Nevada, 9 were hospitalized and. 3 developed HUS	Surveillance systems that aided symptoms reporting and disease tracking were set up in those affected states The media and public health advisories encouraged victims to report to health authorities Nevada laboratories distributed sorbitol MacConkey medium to detect *E. coli* O157:H7
May–July 2011 [11]	Germany (mainly northern Germany, including Hamburg) France (Regions near Bordeaux) 16 European countries in total	Total cases in Europe: 4,075 caes Germany: 1,534 cases identified by June 1 France: 10 cases	O104:H4 possessing features of EAEC and STEC	Contaminated fenugreek sprouts	In Europe generally, the major symptom was bloody diarrhea, 908 (22%) of the confirmed cases were complicated by HUS and 50 deaths were reported In Germany, 470 (31%) developed HUS while 7 in France had this. complication	Several cohort studies and traceback investigations were conducted on restaurants in Germany and France, identifying fenugreek sprouts imported from Egypt as the source. EU-wide ban was subsequently placed on fenugreek seeds and similar products from Egypt
December 2012–January 2013 [78]	3 provinces in Canada	31 total confirmed cases New Brunswick—7 Nova Scotia—11 Ontario—13	*E. coli* O157:H7	24 (83%) ate sandwiches/burgers garnished with lettuce from chain A or B restaurants	13 (42%) victims were hospitalized, 1 (3%) developed, but no deaths were reported	Measures were carried out to identify potential sources of contamination within food service facilities The Canadian Food Inspection Agency (CFIA) conducted a risk assessment for shredded lettuce served at Chains A and B
July 12, 2013–September 29, 2013 [79]	5 Canadian provinces	29 cases	*E. coli* O157:H7	26 (90%) reported eating Gouda milk cheese from a dairy plant in British Columbia	5 patients were hospitalized, while 1 died	The CFIA initiated a food recall program for all raw milk cheese cut packaged by the dairy plant from May 27 to September 17, 2013 to identify the source of infection. The British Columbia CDC prohibited the sale and processing of all raw and pasteurized milk cheese pending implementation of preventive measures
October 7, 2018–December 4, 2018 [18]	16 states in the US and the District of Columbia	62 people infected	*E. coli* O157:H7	Romaine lettuce harvested from the Central Coastal growing regions of northern and central California	25 individuals were hospitalized, of which 2 developed HUS. No deaths were reported	Traceback investigations conducted by FDA and CDC on farms and cooling facilities in california identified the outbreak to be from sediment aggregated within an agricultural water reservoir on Adam Bros. Farming Inc. farm in Santa Barbara County
August 10, 2020–October 31, 2020 [20]	19 states in the US and the District of Columbia	40 people infected	*E. coli* O157:H7	Fresh leafy greens although investigators were unable to identify the specific type or brand of leafy greens implicated	20 individuals were hospitalized, of which 4 developed HUS. No deaths were reported	Epidemiological and traceback studies conducted identified the potential sources. Of 23 people interviewed, 16 ate spinach while 15 ate romaine lettuce. FDA subsequently recommended measures on washing preparation of leafy greens
August 3 to August 16, 2020 [82]	United Kingdom 83% were reported in Midlands region of England and in Wales	36 cases England: 27 Wales: 9	*E. coli* O157:H7	Fast-food Cucumbers 64% confirmed eating in the same restaurant chain	13 victims were hospitalized among which 25 reported bloody diarrhea. No case of HUS was reported	Questionnaires were administered to all new cases within 24 h of notification and reported to the National Enhanced Surveillance System for STEC infection (NESSS) Microbiological investigations were carried out on human and food samples to identify the strains of *E. coli* implicated and suitable antibiotics to administer FSA led food chain investigations with focus on chain A restaurants. A communication system to inform the European Union of the outbreak investigation was set up through the Rapid Alert System for Food and Feed (RASFF)
September 12, 2024–October 21, 2024 [14]	14 states in the US	104 cases	*E. coli* O157:H7	80 (99%) of 81 people interviewed confirmed eating at McDonald's, of which 63 reported a menu garnished with fresh, slivered onions provided by Taylor Farms	34 patients required hospitalizations, 4 developed HUS, and 1 older adult died	The menu items implicated were temporarily withdrawn from sales Federal and state public health agencies like USDA, FDA, and CDC collaborated on measures and investigations to trace and identify the sources of contamination Supply from the Taylor Farms which distributed the contaminated onions was halted until pending investigation findings and implementation of control strategies

### Lessons learned from the outbreaks

#### Rapid response and investigation in outbreaks

Quick coordination among public health agencies is critical in tracking down and managing the source of an outbreak. In these cases, these agencies, including the Public Health Agency of Canada, The Canadian Food Inspection Agency, The Food Standard Agency (FSA) in the United Kingdom, The United States Department of Agriculture (USDA), the Food Safety and Inspection Service, Food and Drug Administration (FDA) and Centers for Disease Control and Prevention (CDC), actively investigated and analyzed traceback data to identify the contaminated ingredient, with frequent updates to the public [52, 82, 84].

Till date, several actions have been taken to investigate *E. coli* outbreaks. These include initiating laboratory testing of suspect food products, patient samples, food handler samples, and environmental samples to validate the presence of pathogenic *E. coli* strains. Molecular subtyping tools like whole genome sequencing (WGS) have been utilized by public health agencies like CDC to identify potential contamination sources and link incidences [85]. Similarly, the FDA and USDA cooperate with food manufacturers to isolate sources of *E. coli* contamination and conduct regular retrospective investigations to map the circulation chain of implicated foods [86]. Food safety bodies like FSA implement risk communication approaches during outbreaks, including sending out recalls, food safety warnings, and advisories to mitigate further exposure. They also ensure that contaminated food products identified are removed from the market [82]. Remarkably, these concerted efforts by these organizations have strengthened disease preparedness and swift response across regions and international borders, ultimately reducing the risks associated with *E. coli* outbreaks.

#### Food safety precautions for businesses and suppliers

McDonald's proactive measures (e.g., stopping the sales of their Quarter Pounders and slivered onions) [14] demonstrate that early removal of potentially contaminated products can reduce further risks. Additionally, Taylor Farms’ voluntary recall of their onion products [14] is a key example of supplier responsibility in outbreak management. Fast-food chains and suppliers play a crucial role in outbreak prevention by maintaining strict food safety standards and responding promptly to potential contamination [84]. Importer/Processor X had to initiate a precautionary recall for shredded lettuce items supplied to Chain A and Chain B restaurants during the E. coli outbreak in Canada [81].

#### E. coli as a public health concern

The *E. coli* outbreak emphasizes the public health risks associated with certain strains like O157, which can lead to serious health complications, including HUS [48, 53]. The low infectious dose of these strains means that a minute number of microbes can trigger severe illness. Other STEC strains are implicated for bloody diarrhea, renal failure, and long-term health complications like hypertension and chronic kidney disease [14].

The widespread presence of *E. coli* in food supply chains even worsened these public health concerns. Factors such as cross-contamination during food processing, poor hygiene practices, and inappropriate cooking of high-risk products persistently predispose to outbreaks within communities [52]. These higher risks of severe outcomes from infection disproportionately affect vulnerable groups such as the aged, young children, and immunosuppressed people [75].

### Preventive measures: industry and consumer responsibility

All hands must be on deck both within the fast-food industry and level of consumers to reduce the probability of another *E. coli* outbreak. Concerted efforts ranging from interventions made by fast food restaurants to programs aimed at improving consumer behavior are great steps toward preventing another *E. coli* outbreak.

- Industry responsibility: Fast-food restaurants must adopt strict hygiene and food safety protocols at every stage of food preparation. Systems like HACCP must be fully implemented. For instance, routine monitoring of cooking temperatures to eliminate *E. coli* bacteria in raw foods will reduce contamination risks [67]. Staff should be trained regularly on hygiene practices, such as handwashing, and proper use of gloves. Additionally, periodic audits and inspections of fast-food facilities must be ensured to guarantee compliance with food safety standards and detection of subtle gaps in equipment sanitation, food storage, and waste disposal [68].
- Consumer responsibility: The adoption of these safe food-handling practices and making informed choices will be pivotal in reducing the risk of future *E. coli* outbreaks. Fast-food consumers must thoroughly wash hands before and after every meal [82], also ensuring that food items, especially meats, are cooked to recommended temperatures to disarm *E. coli* [67]*.* Ultimately, the decision to choose fast-food joints that prioritize food safety, sustain transparent food-handling practices, and adhere to food safety regulations must consistently be made by consumers [21].

By complementing the aforementioned industry-centered interventions with consumer education and actions, the risks of subsequent *E. coli* outbreaks in the future can be minimised, ensuring safer fast-food systems and improved health outcomes among the public.

### Quality and microbiological criteria for raw and ready-to-eat (RTE) products

Food safety regulatory agencies that strengthen disease prevention have also formulated microbiological criteria to reduce the risk of *E. coli* contamination in raw and RTE edible materials.

For example, the Codex Alimentarius Commission (CAC), an international food standards organisation unitedly founded by the FAO and the WHO in 1963, advocates that raw meat the acceptable *E. coli* level must not exceed 10 colony-forming units per gram (CFU/g), while the absence of *E. coli* O157:H7 and other STEC in RTE products must be assured to ensure consumer safety [56].

The microbiological criteria for RTE foods set by the European Union Regulation (EC) No. 2073/2005 also recommend the absence of STEC in 25g of a product due to the severe health risk posed by these strains [87]. Similarly, the United States’ Food Safety and Inspection Service (FSIS) introduced actions designed to reduce *E. coli* O157:H7 contamination of meat and meat products, particularly raw ground beef. These including instituting regulations enforcing the installation of safe handling label on raw meat packages, expanding its microbiological testing program to incorporate *E. coli* O157:H7 in raw ground beef, promulgating HACCP Systems regulations, and enforcing “zero tolerance” for visible fecal contamination on carcasses at slaughter by the food inspection program committee. These measures significantly reduced the incidence of *E. coli*-mediated foodborne illnesses in the US [88].

### Fast-food industry interventions

A study conducted in Canada reported that improved interventions by food regulatory agencies on beef suppliers reduced the prevalence of *E. coli* infection [89]. Fast-food restaurants are responsible for ensuring that meat is obtained from beef processing plants that fully comply with health regulations.

There is data to link poor hygiene compliance and inadequate cooking and refrigeration to the spread of food-borne diseases, including *E. coli* infections. Poor hygiene by the fast-food workers should also be shunned. A study by the Environmental Health Specialists Network (EHS-Net) determined that one of the food safety hazards was ill and infected food workers reporting for duty because they did not want coworkers staffed to be overworked, and they were also afraid of losing their jobs [90]. Infected food workers have been identified as contributors to over 80% of food-borne infection outbreaks [90].

### Consumer education and awareness

The increase in food-borne diseases has been associated with consumers' lack of knowledge of these infections. Evidence suggests that many food-borne illnesses can be traced to improper food handling and consumption practices [91]. A study reported that consumers have an optimistic bias toward food-borne diseases [92]. This behaviour makes consumers overly optimistic and neglects health preservation attitudes and practices [92].

Effective educational campaigns, such as the USDA's "Clean, Separate, Cook, Chill" initiative, have proven successful in teaching consumers fundamental food hygiene practices to minimize risks when handling food (Health and Safety). Mason (2024) also demonstrated that educational interventions significantly enhance consumer awareness and behavior, ultimately reducing foodborne illnesses [93].

Consumers ought to be taught to identify the risk levels of whatever food they consume from fast-food restaurants. This would enable them to make informed choices about their food choice, restaurant and food-handling behavior. Increasing consumer awareness of food-borne pathogens may positively reduce foodborne diseases [94].

## Conclusions

The fast-food industry is a rapidly growing business with a worldwide consumer base. This is mostly due to urbanization and the fact that people do not have the time to prepare homemade meals. Hence, there is an ever-increasing need for fast-food chains to take proactive safety measures to ensure their customers' health.

Health and safety regulations for fast-food operators should be stringent to prevent loopholes facilitating pathogen contamination and disease transmission. Meat used for cooking must be cooked adequately and obtained from farms/suppliers with necessary safety and health clearance. Other ingredients used to prepare these fast foods should be cultivated and processed under the best hygienic conditions. In the event of an outbreak, restaurants should immediately suspend or change their raw produce suppliers pending further investigations by the appropriate authorities.

Restaurants with bad hygiene and safety ratings should be closed indefinitely. Meanwhile, sick workers should not be allowed to work in restaurants until they fully recover. Restaurant workers should be strongly discouraged from practicing poor hygiene. To reduce cross-contamination, restaurant workers should receive compulsory training on hygienic procedures and be supervised by kitchen managers certified in food safety.

Finally, consumers should be properly educated on the prevalence of food-borne illnesses and the importance of taking preventative measures to protect themselves. Consumer education and awareness programs should specifically target people less likely to be aware of food-borne pathogens as a safety problem and thus may practice fewer food-handling behaviors.

## Data Availability

No datasets were generated or analysed during the current study.

## Abbreviations

CDC: Centers for Disease Control and Prevention
DAEC: Diffusely Adherent *Escherichia coli*
EAEC: Enteroaggregative *Escherichia coli*
EIEC: Shigella/Enteroinvasive *Escherichia coli*
EPEC: Enteropathogenic *Escherichia coli*
ETEC: Enterotoxigenic *Escherichia coli*
STEC: Shiga toxin-producing *Escherichia coli*
FDA: Food and Drug Administration
FIORP: Foodborne Illness Outbreak Response Protocol
FSA: Food Standard Agency
HACCP: Hazard Analysis Critical Control Point
HUS: Hemolytic Uremic Syndrome
USDA: United States Department of Agriculture
